# Symptoms as the main problem: a cross- sectional study of patient experience in primary care

**DOI:** 10.1186/s12875-016-0429-8

**Published:** 2016-03-10

**Authors:** Marianne Rosendal, Anders Helles Carlsen, Mette Troellund Rask

**Affiliations:** Department of Public Health, Research Unit for General Practice, Aarhus University, Bartholins Alle 2, DK-8000 Aarhus C, Denmark

**Keywords:** MESH: signs and symptoms, primary health care, general practice, cross-sectional, patient satisfaction, treatment outcome, NON-MESH: medically unexplained symptoms

## Abstract

**Background:**

Symptoms are common in primary care. Besides providing thorough assessment of possible severe disease, the general practitioner (GP) must ensure good health care to all patients, irrespective of diagnoses. We aimed to explore patient satisfaction with the provided care and how well expectations in patients were met when no diagnosis was made during the consultation.

**Method:**

Cross-sectional study based on a questionnaire survey conducted in 2008–2009 among 377 GPs and their patients in the Central Denmark Region. A total of 2286 patients completed a questionnaire after the consultation (response rate: 54 %). The questionnaire included four satisfaction items from the EUROPEP instrument and a question about unmet expectations. For each patient, the GP answered a one-page registration form including information about the main problem in the consultation, chronic disorders and assessment of prognosis. Statistical analyses were adjusted for patient characteristics and GP clustering.

**Results:**

A higher proportion of patients reported illness worry (20 vs. 17 %, *p*-value: 0.005), unmet expectations (17 vs. 13 %, *p*-value: 0.019) and dissatisfaction with their GP after the consultation when no diagnosis was made. Dissatisfaction was primarily related to the medical examination (adjusted OR 1.30; 95 % CI: 1.06–1.60) and GP explanations (adjusted OR 1.40; 95 % CI: 1.14–1.71). Exploratory analyses revealed an association between dissatisfaction with examination and the GP assessment that symptoms were unrelated to biomedical disease. This association was found both in patients with ‘symptoms only’ and patients given a specific diagnosis.

**Conclusion:**

GPs are challenged by patients presenting symptoms that do not fit the patterns of biomedical diagnoses. The current study demonstrates more illness worry, unmet expectations and dissatisfaction with the consultation in these patients compared to patients receiving a diagnosis. This trend is true for all patients assessed as having ‘symptoms only’ at the end of a consultation and not only for the minority group with ‘medically unexplained symptoms’. As primary care is the frontline of the health-care system, symptoms are managed as the main problem in almost one in three consultations. It is about time that we take the same professional approach to symptoms as we have done for years to biomedical disease.

**Electronic supplementary material:**

The online version of this article (doi:10.1186/s12875-016-0429-8) contains supplementary material, which is available to authorized users.

## Background

General practitioners (GPs) manage symptoms as the main problem in a third of all health-related consultations [1]. In many cases, no specific diagnosis is reached because the GP has not yet finished the assessment or because the patient simply has symptoms as such. The medical literature on symptoms has primarily focused on persistent and disabling symptoms without biomedical explanations, i.e. medically unexplained symptoms (MUS). However, MUS represent only part of a much larger spectrum of patients presenting symptoms in primary care. This paper targets the whole spectrum of patients with symptoms as the main problem in the consultation.

Patient satisfaction with care is an important parameter in health care; it is a criterion for good health care [2] and may also be associated with improved outcome. Several studies have demonstrated an association between satisfaction with care and communication [3]. A few studies also indicate higher chance of symptom alleviation, functional improvement and less post-visit worry when the patients experience good communication, i.e. receive adequate diagnostic and/or prognostic information [4–7].

Most patients are generally highly satisfied with their GP [8]. Nevertheless, studies on patients with MUS have demonstrated lower satisfaction with health care compared to patients labelled with a biomedical diagnosis [9, 10]. Satisfaction with care in patients with MUS has been shown to correlate with GP communication [4, 11] for specific content-related aspects of care, e.g. being taken seriously by the GP or receiving clear information regarding the treatment [9]. At the same time, GPs tend to experience frustration and difficulty when dealing with MUS [12, 13] or dissatisfied patients [5, 14]. Consequently, there seems to be increased risk of conflict, poor communication and dissatisfaction when patients consult their GP with symptoms for which no immediate diagnosis can be reached; this also includes cases for which symptoms cannot yet be characterised as ‘medically unexplained’. Although current evidence indicates lower satisfaction among patients with MUS and a considerable association between patient satisfaction and GP communication, little knowledge exists on symptoms as the main problem in the consultation. As exploration of the patient experience may lead to the identification of specific areas in need of change when dealing with symptoms in general, we aimed to explore the patients’ experience with the GP consultation to identify patient- and GP-related factors that may be associated with poor experience. Furthermore, we aimed to evaluate whether patients who did not receive a diagnosis for any specific disease/disorder during the consultation had a different experience than patients who did receive a specific diagnosis. The patient experience was measured in two ways: 1) satisfaction with different aspects of care and 2) whether or not the patient’s overall expectations were met.

## Methods

### Design and setting

The cross-sectional study was based on a survey of Danish general practice conducted from December 2008 to December 2009 [15]. The Central Denmark Region is a mixed rural and metropolitan area with almost 1.3 million inhabitants served by 871 GPs (covering approximately 20 % of the entire Danish population). The Danish health-care system is tax-funded, and 98 % of all Danes are listed with a specific general practice.

### Participants

All GPs in the Central Denmark Region were invited to participate. GPs who had first signed a written consent to participate registered all patient contacts during one randomly assigned work day. The GPs received remuneration for their participation (EUR 32) and for each registered contact (EUR 3).

**Fig. 1:**
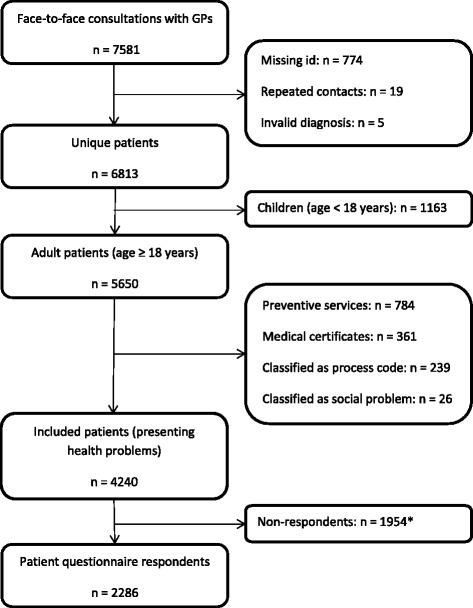
Flow of patients. *) Of these, 1552 did not answer the posted questionnaire; 392 had obtained research protection, 7 had died since the GP visit and 3 had no valid address

For the purpose of the present paper, we included all identifiable patients aged ≥ 18 years who had visited their GP because of a health problem and completed a questionnaire approximately two weeks after the consultation. Patients who had visited their GP because of ‘preventive health services’ or ‘other problems’ (e.g. social issue or para-clinical testing) and patients who had received a home visit were excluded (Fig. 1). Only the first contact was included if a patient appeared more than once in the collected data (Fig. 1). We compared patients for whom the GP could not provide a specific diagnosis at the end of the consultation (‘symptoms only’) with patients who received a specific diagnosis.

### Data sources, variables and outcome measures

Background information on patients listed with participating practices was obtained from the regional section of the Danish National Health Service Register and Statistics Denmark.

Each patient contact was registered on a one-page registration form by the GP (Additional file 1). The form included the civil registration number (unique Danish personal identification number) and information on gender, age, type (face-to-face consultation, home visit, telephone consultation or e-mail consultation) and content of encounter (health problem, preventive care or health certificate, e.g. for driver’s license or life insurance). The GP also stated the main problem as assessed at the end of the consultation. The information could be entered as free text or as International Classification of Primary Care (ICPC) codes. Text stated in the registration forms was translated into ICPC-2-R codes by an ICPC-trained medical student using standard terminology [16]. The ICPC classification system consists of 17 chapters, each of which is divided into seven components: symptom codes (component 1), process codes (components 2–6) and specific disease codes (component 7). All entered ICPC codes were reviewed by a health-care project manager and dichotomised into two patient groups: ‘symptoms only’ (component 1, except for addiction and contraception) and ‘specific diagnoses’ (other codes). Patients registered with main health problems coded as ‘social issues’ were excluded from the analyses.

Additional information on chronic conditions and how the GP assessed the final outcome to be in due time (specific disease, resolving symptom or persistent symptom/MUS) was also extracted from the GP registration form. For the analyses, the GP–assessed outcome was dichotomised into ‘disease’ or ‘no disease’; the latter including both resolving and persistent symptoms.

The questionnaire sent to patients (Additional file 2) included questions on subjective health (SF-12) and a single item on worry phrased: ‘During the past 4 weeks, how much have you been bothered by worries that there is something seriously wrong with your body?’ From the SF-12, the physical component summary (PCS) score and the mental component summary (MCS) score were calculated using US norms. The ‘worry question’ was dichotomised into ‘no worry’ (not at all, a little) and ‘worry’ (some, quite a lot, a lot).

Our primary outcome was patient satisfaction with care. We used items from the EUROPEP instrument [8] on 1) the doctor-patient relationship, 2) medical care and 3) information and support. In order to keep the questionnaire brief, only the following four EUROPEP items were included [17]: What is your opinion of the general practitioner after this visit with respect to 1) making you feel you had time during the consultation, 2) thoroughness, 3) physical examination of you and 4) explaining the purpose of tests and treatments. Due to ceiling effect and highly skewed distributions, all items were dichotomised into ‘dissatisfied’ (bad, fair, good) and ‘satisfied’ (very good, excellent). Furthermore, a number of items were coded as missing because the respondent had answered ‘not relevant/don’t know’. The EUROPEP questions were supplemented with a question about expectations: Were your expectations met? This question was dichotomised into ‘no’ (not at all, a little, somewhat) and ‘yes’ (a good deal, completely).

Patients were informed about the project in writing when receiving a questionnaire after their GP visit, and patients consented to participate by answering and returning the questionnaire. As an approval had been obtained from the Danish Health and Medicines Authority (J.no. 7-604-04-2/49/EHE), the participating GPs could report data for research on all patients without patient consent. The project was approved by the Danish Data Protection Agency (J.no. 2010-41-5671). According to Danish law, approval from the regional committee on health research ethics was not needed for this type of study.

### Statistical methods

In the analyses of patient characteristics, GP assessments and the association between patient expectations and patient satisfaction with care, the estimates were adjusted for patient age, gender and presence of chronic disorder (yes/no). Statistical analyses were performed using generalised linear models (GLM) with id link and the Bernoulli family including robust variance estimation to account for clustering at GP level supplemented by Wald tests in combined analyses of educational level. In crude analysis of patient age and gender Student’s t-test and chi-square test were used.

In the explorative analyses of patient expectations and patient satisfaction with care, tests were mutually adjusted for a number of patient-related factors (gender, age, education, GP-reported chronic disorder, worry and subjective health, i.e. the PCS and MCS scores of the SF-12) and a number of GP-related factors (gender, years of practice experience and practice type). Differences in satisfaction with care between patient groups were analysed using two regression models for calculation of odds ratios (ORs): 1) univariate logistic regression and 2) multivariate logistic regression, which were adjusted for possible confounders identified in the initial explorative analyses (i.e. patient gender, age, education, chronic conditions, subjective health (PCS and MCS) scores and worry). These analyses were performed using GLM as described above.

Each GP registration form and patient questionnaire was optically scanned by Teleform 8.0, and Stata 13.1 [18] was used for the statistical analyses.

## Results

The survey included 377 of the 871 invited GPs (43 %). Participating GPs did not differ from GPs in the catchment area with regard to type of practice, but we found higher representation of female GPs (*n* = 167, 44.3 % of participants) and lower representation of GPs with more than 20 years of practice experience (*n* = 85, 22.6 % of participants). Patients listed with participating GPs were comparable to those listed with non-participating GPs with regard to age and gender distribution [19].

### Patient characteristics

A total of 4240 patients with face-to-face contacts about health problems were invited to participate in the present study (Fig. 1). Of these, 2286 (54 %) answered the patient questionnaire and were included.

Non-respondents differed from respondents on several aspects. Non-respondents were younger (*p* < 0.001), had fewer years of education (*p* < 0.001), more were living alone (*p* < 0.001) and fewer had a chronic disease (*p* = 0.012). We found no differences with regard to gender (*p* = 0.463) and frequency of ‘symptoms only’ (*p* = 0.159).

The characteristics of patients with ‘symptoms only’ and patients with a specific diagnosis are shown in Table 1. Patients with ‘symptoms only’ were slightly younger, more were women and fewer had concurrent chronic disorders.

**Table 1:** Characteristics of patients with ‘symptoms only’ compared to patients with specific diagnoses

	Symptoms (n, %)	Specific diagnoses (n, %)	*P*-value
Number of patients, n (%)	796 (34.8)	1490 (65,2)	
Gender, male, n (%)	275 (34.6)	582 (39.1)	**0.034**
Age, mean (SD)	53.2 (17.7)	54.8 (17.2)	**0.036**
Education			
≤ 10 years	241 (31.0)	494 (33.8)	0,275*
10–15 years	370 (47.6)	702 (48.1)	
> 15 years	166 (21.4)	264 (18.1)	
Cohabiting (vs. single)	578 (72.6)	1113 (74.8)	0,286**
Physical functioning (SF-12, PCS), mean (SD)	44.5 (11.8)	44.1 (12.1)	0,437**
Mental functioning (SF-12, MCS), mean (SD)	48.0 (9.9)	48.6 (10.2)	0,137**
Illness worry	156 (20.1)	243 (16.7)	0.005**
GP assessments			
Chronic disorder	356 (44.7)	779 (52.3)	0.003**
Final outcome expected to be ‘no disease’	362 (46.5)	272 (18.9)	<0.001**

Notes

*Wald test

**Adjusted for patient age, gender and chronic disorders

Numbers in bold indicate statistical significance

More patients with ‘symptoms only’ than patients with a specific diagnosis (20 vs. 17 %) were worried that something was wrong with their body (Table 1). Nevertheless, the GPs did not suspect the presented symptoms to be signs of disease or to develop into disease in half (71 of 154) of these patients.

### Factors associated with patient experience

We explored correlations between (on the one hand) dissatisfaction and unmet expectations and (on the other hand) patient characteristics, GP assessments and GP characteristics in both patient groups (Table 2). The mutually adjusted analyses showed higher probability of dissatisfaction in both patient groups for most aspects when the subjective health scores were in the lower range. This was also true for younger age. Specifically with regard to GP examination, more patients were dissatisfied when the GP assessed that the symptoms would remain symptoms or resolve.

**Table 2:** Mutually adjusted analyses of dissatisfaction and unmet expectations according to GP assessment of diagnosis

		Dissatisfaction with												Unmet expectations		
		Time			Thoroughness			Examination			Explanation					
		(OR, 95% CI)^a^			(OR, 95% CI)^a^			(OR, 95% CI)^a^			(OR, 95% CI)^a^			(OR, 95% CI)^a^		
***Patients with ‘symptoms only’*** *(n)*		660			656			608			631			659		
Patient characteristics:																
	Male (vs. female)	0.82	0.58	1.16	1.02	0.72	1.45	0.84	0.59	1.21	**0.66**	**0.46**	**0.96**	1.08	0.68	1.73
	Age	1.00	0.98	1.01	0.99	0.98	1.00	0.99	0.98	1.00	**0.99**	**0.97**	**1.00**	**0.99**	**0.97**	**1.00**
	Education 10–15 years (vs. <10 years)	0.96	0.66	1.39	0.97	0.65	1.46	1.06	0.73	1.54	1.12	0.76	1.66	0.97	0.59	1.60
	Education >15 years (vs. <10 years)	0.80	0.50	1.29	0.83	0.50	1.38	0.82	0.49	1.35	0.81	0.49	1.32	**0.47**	**0.22**	**0.99**
	Chronic disorder (vs. none)	0.71	0.50	1.01	**0.67**	**0.47**	**0.94**	0.70	0.48	1.03	0.80	0.56	1.16	0.92	0.58	1.46
	Physical functioning (SF-12, PCS)	**0.98**	**0.96**	**1.00**	**0.98**	**0.96**	**0.99**	**0.97**	**0.95**	**0.98**	**0.98**	**0.96**	**0.99**	**0.97**	**0.95**	**0.99**
	Mental functioning (SF-12, MCS)	0.99	0.97	1.01	0.99	0.97	1.01	0.98	0.96	1.00	**0.98**	**0.96**	**1.00**	0.98	0.96	1.00
	Illness worry	0.87	0.55	1.38	1.07	0.68	1.68	0.85	0.54	1.32	0.88	0.56	1.38	1.61	0.95	2.73
GP assessment:																
	Expect outcome to be ‘no disease’	1.12	0.80	1.57	1.25	0.88	1.77	**1.57**	**1.11**	**2.22**	1.00	0.71	1.42	1.32	0.86	2.04
GP characteristics:																
	Male (vs. female)	1.01	0.70	1.45	0.89	0.60	1.30	0.97	0.65	1.43	1.04	0.71	1.54	1.06	0.65	1.73
	Years in practice	1.00	0.97	1.02	1.00	0.98	1.02	0.99	0.97	1.02	1.00	0.97	1.02	0.98	0.96	1.01
	Practice type solo (vs. partnership)	0.92	0.60	1.41	1.01	0.65	1.59	1.01	0.65	1.56	1.01	0.68	1.51	1.02	0.58	1.81
***Patients with a specific diagnosis*** *(n)*		1212			1209			1113			1146			1214		
Patient characteristics:																
	Male (vs. female)	0.90	0.68	1.19	0.94	0.72	1.23	1.21	0.92	1.58	0.95	0.71	1.25	0.86	0.59	1.24
	Age	0.99	0.98	1.00	**0.99**	**0.98**	**1.00**	**0.99**	**0.98**	**1.00**	0.99	0.98	1.00	**0.98**	**0.97**	**0.99**
	Education 10–15 years (vs. <10 years)	1.02	0.79	1.32	0.87	0.66	1.14	1.07	0.78	1.47	1.12	0.85	1.49	0.80	0.55	1.17
	Education >15 years (vs. <10 years)	0.79	0.55	1.14	0.75	0.51	1.10	0.88	0.58	1.33	0.79	0.58	1.08	**0.57**	**0.33**	**0.97**
	Chronic disorder (vs. none)	0.86	0.64	1.16	0.86	0.64	1.15	0.97	0.73	1.30	0.98	0.97	1.00	1.05	0.73	1.50
	Physical functioning (SF-12, PCS)	**0.99**	**0.97**	**1.00**	0.99	0.98	1.00	0.99	0.98	1.00	**0.98**	**0.97**	**1.00**	**0.98**	**0.97**	**0.99**
	Mental functioning (SF-12, MCS)	**0.99**	**0.97**	**1.00**	**0.99**	**0.97**	**1.00**	**0.98**	**0.97**	**1.00**	**0.98**	**0.97**	**1.00**	0.98	0.96	1.00
	Illness worry	1.28	0.91	1.81	1.30	0.91	1.87	1.36	0.92	2.01	1.15	0.78	1.70	**1.68**	**1.09**	**2.57**
GP assessment:																
	Expect outcome to be ‘no disease’	1.13	0.85	1.50	1.12	0.84	1.50	**1.45**	**1.04**	**2.02**	1.38	1.00	1.91	1.24	0.82	1.88
GP characteristics:																
	Male (vs. female)	1.03	0.77	1.36	1.06	0.80	1.41	1.03	0.74	1.42	1.09	0.80	1.47	0.91	0.61	1.36
	Years in practice	1.00	0.98	1.01	0.99	0.98	1.01	1.00	0.98	1.02	1.01	0.99	1.02	0.99	0.97	1.01
	Practice type solo (vs. partnership)	0.97	0.71	1.32	0.93	0.68	1.27	1.12	0.76	1.66	0.99	0.69	1.41	0.90	0.53	1.54

Notes

^a^Analyses are mutually adjusted

Numbers in bold were found to be statistically significant

High values on PCS and MCS scores indicate better health

For patients with ‘symptoms only’, more women than men were dissatisfied with the explanation provided by the GP, and more patients with concurrent chronic disorders were satisfied with GP thoroughness compared to patients without chronic conditions.

In line with our finding on satisfaction with care, young age and low subjective health scores were associated with unmet expectations in both patient groups. Furthermore, fewer years of education and high illness worry were also associated with higher probability of not having expectations met.

GP gender, years of practice experience and practice type were neither associated with satisfaction nor with expectations.

### Patient experience according to GP assessment of diagnosis

After statistical adjustments for the potential confounders described above, we found that a higher proportion of patients with ‘symptoms only’ reported dissatisfaction and unmet expectations compared to patients given a specific diagnosis. Half of the patients with ‘symptoms only’ were dissatisfied with at least one item compared to 44 % of patients with a specific diagnosis (OR 1.26 (1.06-1.49) and adjusted OR 1.26 (1.05-1.51)). The higher rate of dissatisfaction among patients with ‘symptoms only’ was present on all of the four parameters, but only reached statistical significance with regard to GP examination and explanation when compared to patients with a specific diagnosis (Table 3).

**Table 3:** Satisfaction with care and unmet expectations in patients with ‘symptoms only’ vs. patients with specific diagnoses

	Symptoms only	Specific diagnosis	Unadjusted	Adjusted^a^
	n (%)	n (%)	OR (95% CI)	OR (95% CI)
Dissatisfied with time	291 (37.1)	498 (34.0)	1.14 (0.95–1.37)	1.15 (0.95–1.39)
Dissatisfied with thoroughness	257 (33.0)	442 (30.4)	1.13 (0.93–1.36)	1.11 (0.91–1.36)
Dissatisfied with examination	261 (36.5)	414 (31.0)	**1.28 (1.05–1.55)**	**1.30 (1.06–1.60)**
Dissatisfied with explanations	268 (35.9)	393 (28.6)	**1.40 (1.15–1.69)**	**1.40 (1.14–1.71)**
Expectations not met	131 (16.7)	195 (13.4)	**1.30 (1.02–1.64)**	**1.37 (1.05–1.78)**

Notes

^a^Adjusted for patient age, gender, education, chronic disorders, subjective health scores (PCS and MCS) from SF-12, illness worry and GP clusters

Numbers in bold were found to be statistically significant

## Discussion

### Summary

A higher proportion of patients with ‘symptoms only’ reported illness worry (1 in 5), unmet expectations (1 in 6) and dissatisfaction with at least one of four satisfaction measures (1 in 2) after the consultation compared to patients with a specific diagnosis. Comparisons of the two patient groups also revealed a higher frequency of dissatisfaction specifically with GP examination and explanation in patients with ‘symptoms only’ (1 in 3). Furthermore, the GP assessment that symptoms remained symptoms or would resolve was associated with dissatisfaction with examination irrespective of whether or not the patient was diagnosed with a specific disease.

### Strengths and limitations

A major strength of this study was the large number of GPs and patients who agreed to participate. However, substantial numbers of patients chose not to respond to the questionnaire. Consequently, the generalisability may be compromised for young people with fewer years of education and no chronic disease who live alone. As these are all factors which would potentially contribute to dissatisfaction, non-response would tend to bias our results towards lower frequencies and lower differences between patient groups. The setting (Denmark, Western Europe) itself is selected, and we do not know whether the results may apply to other primary-care settings.

The study was strengthened by the linkage between GP registrations and patient questionnaires as this procedure ensured that patients could be linked to the individual GPs rather than to the practices. Furthermore, the short period of time between consultation and completion of patient questionnaire ensured that both ‘satisfaction with care’ and ‘patient expectations’ were associated with the specific health problem managed by the GP during the consultation. However, no causal assumptions can be made from results based on exploratory analysis (here: associations between patient experiences and patient/GP factors), but this is true for all cross-sectional studies. Results might also change over time, for example due to spontaneous symptom alleviation as seen in a previous study with three months of follow-up [20]. We do not know how time itself may have affected the differences between the patients who were given a diagnosis for their health problem and the patients who were not.

GP diagnostics have shown large variability, especially for MUS [21]. In the present study, the GP diagnoses were not externally validated. While studies of GP inter-rater variability have demonstrated substantial variations at the level of diagnostic codes (agreement: 56 %), the variations at the level of components (symptom vs. disease) are generally smaller (agreement: 70 %) [22]. Misclassification at the level of components would most likely moderate results towards smaller differences between groups because we would expect more patients with ‘symptoms only’ to be misclassified as specific disorders than the other way around, both because GPs generally exhibit biomedical preference [19] and because some ICPC categories for diseases include functional somatic syndromes.

The most important outcome measure in this study was based on EUROPEP, which is an internationally validated tool developed specifically for measuring satisfaction with care in general practice [8]. However, three problems emerged. Firstly, the EUROPEP lacks a factorial model across items [17], and analyses were based on single items. Secondly, the instrument has a problem with ceiling effect, i.e. most patients are very positive in their evaluation of their GP [17]. In order to reduce this problem, we chose to dichotomise responses at the high end on the Likert scale in line with previous studies [23]. This resulted in acceptable numbers for further statistical analyses. Thirdly, as the number of items in the patient questionnaire was constrained, we included only a few of the EUROPEP items and one item focusing on expectations. Thus, we were unable to further explore issues relating to patient satisfaction, expectations and communication.

### Comparison with existing literature

Direct comparison between studies in the field is hampered by large variations in outcome measures and follow-up. Half of the patients presenting common symptoms in one US primary care study were not fully satisfied, and 30 % had unmet expectations [5, 24]. However, another study found that only 12 % had at least one unmet expectation [25]. In a previous Danish primary care study using the complete version of the same questionnaire with different cut-points and duration of follow-up (one year), 60-75 % of the included patients were not completely satisfied [26]. These figures correspond largely to the dissatisfaction of 50 % reported in our study for at least one aspect of the EUROPEP items and the 17 % that we identified for unmet expectations.

Our finding of a high frequency of dissatisfaction among patients with ‘symptoms only’ is equivalent to the findings for MUS, where up to 29 % of patients reported to experience dissatisfaction [9]. Specifically, we found a correlation between GP assessment that symptoms were unlikely to be signs of disease and low patient satisfaction with the GP examination. In line with this, Palmer found that 20 % of the dissatisfied patients with upper limb pain reported that the GP had not examined thoroughly enough [27].

Our finding of dissatisfaction with explanations is in line with recent research emphasising the importance of providing adequate explanatory models to patients, particularly when no immediate diagnosis can be made. It is argued that ‘explanations are a necessary counterweight to the power of diagnostic testing and negative results. The rational explanation, while imperfect, makes sense to both doctor and patient and promotes appropriate action’ [28]. Hence, communication of tangible explanations may hold the potential to improve patient satisfaction and possibly also health outcomes when no diagnosis can be made. Our results are generally in agreement with the current evidence for persistent MUS, but the implications identified in the present study apply broadly to all the symptoms presented in general practice.

We examined the degree to which patients felt that their expectations were met, but we did not explore which kinds of expectations were met or not met. According to the literature, diagnostic and prognostic information plays a role with regard to expectations, but this information is not always communicated during the consultation [6]. Failure to communicate, for example, diagnostic information to patients with ‘symptoms only’ was also a key issue in our study as we found considerable dissatisfaction with explanations in this patient group. The fact that patients have unmet expectations is important as fulfilled expectations have been reported to correlate with later symptom alleviation, functional improvement and health care use [6, 25]. The question as to whether improved information relating to explanatory models, coping and prognosis may improve patient satisfaction and ultimately provide better patient health needs further exploration.

Our exploratory finding that better (mental and physical) health, older age and chronic conditions correlated with higher satisfaction is identical to previous findings on patient-related factors [6, 20, 27, 29, 30]. We found no correlations for factors related to GP (gender, age and experience) or type of practice, although previous studies found that GPs with more than five years of working experience in solo practices were more comfortable with the management of MUS [31]. GP gender and age have also formerly shown associations with satisfaction measures [23, 32], but the reported differences may be caused by variations between studies in regard to patient populations and questionnaire items.

Finally, when no diagnosis was made, 20 % of patients still worried that something was wrong with their body. This number is lower than the 64 % reported by Jackson [5], but the association with unmet expectations was comparable to a previously reported OR of 2.4 (95 % CI 1.5–4.0) for worry when expectations were not met [6]. We do not know how illness worry and expectations influence each other, but the patient experience may be improved if both patient worry and expectations are better tackled by the GP.

## Conclusion

Detection and management of acute and chronic diseases are undoubtedly key tasks for general practice. Patients fitting into this biomedical model are given a diagnosis, which entails that they have a better chance of having their expectations met, feeling satisfied with their GP and not worrying too much about their health than the patients who simply have symptoms (both when entering and leaving the consultation room). This does not imply that every patient should be given a diagnosis, but all patients should be provided with explanations, health-care legitimacy and a basis for action even when they have ‘symptoms only’.

Our results emphasise a need to prioritise and improve the professional approach to symptoms as such in frontline health care. The post-consultation worry, unmet expectations and dissatisfaction among patients, specifically with GP explanation and examination, indicate that patients with symptoms (but no diagnosis) are not taken as seriously as other patients.

Despite the emphasis placed on communication skills in general medicine through many years, evidence indicates a possible benefit from better acknowledgement of symptoms and patient concerns combined with the provision of tangible explanations. As approximately one third of all patients presenting a health problem to the GP have ‘symptoms only’, clinical training and research should focus more on this area to ensure that patients are empowered to deal with their health problem, regardless of diagnostic criteria.

## Additional files

Additional file 1: GP Registration Form. (DOCX 24 kb) Additional file 2: Patient Questionnaire. (DOCX 26 kb)

## Abbreviations

CI: Confidence interval
EUR: Euro
EUROPEP: European patients evaluate general practice
GLM: Generalized linear models
GP: General practitioner
ICPC: International Classification of Primary Care
MCS: mental component summary
MUS: medically unexplained symptoms
OR: odds ratio
PCS: physical component summary
SF: short form
