# Supplementary material for: Symptoms as the main problem: a cross- sectional study of patient experience in primary care
Source: BMC Fam Pract. 2016 Mar 10;17:29. doi: 10.1186/s12875-016-0429-8 (PMC4785648; doi:10.1186/s12875-016-0429-8)
Supplement: Additional file 1: — GP Registration Form. (DOCX 24 kb) [file 12875_2016_429_MOESM1_ESM.docx]

# GP Registration Form

This is a one-way translation of an original Danish survey questionnaire used for general practitioners (GPs).
The original questionnaire was designed in A4 format size in Teleform; the layout was different, but the items are identical. The questionnaire came with instructions (on a separate sheet) for each item.

**Background information**

1. Personal registration number:

Known chronical diseases:

1. 4.
2. 5.
3. 6.

**Patient’s entry to the consultation** (tick A, B and/or C)

1. **Standard consultation** (tick only one box)

□ Face-to-face consultation

□ Telephone consultation

□ Home visit
□ E-mail consultation

(continues at 2 below)

1. **Scheduled preventive health consultation**□ Medical examination/vaccination of child

□ Health prevention - pregnancy

□ Pap-smear test (invited)

□ Vaccination (influenza, travel, etc.)

□ Prearranged health prevention consultation

□ Detection of sexual partners in connection with chlamydia

1. **Contact regarding medical prescription, certificate, etc.**

□ Prescription renewal by telephone/mail

□ Social-medical certificate

□ Sick note/absence certificate

□ Medical certificate for driver’s license

□ Medical certificate for insurance

□ Other medical certificate

Registration of B and C ends here UNLESS the problem requires an additional regular consultation during the same day involving further diagnostic investigations or similar. (Continue at 2 below if the problem requires an additional regular consultation).

**Content of encounter**

2. Primary reason for encounter:

3. Main symptom/diagnosis of encounter:

4. Does the encounter regard A new episode □ Follow-up □

5. Content of encounter (several boxes may be ticked):

□ Prescription of medication/issue of prescription
□ Feedback on test results, etc.
□ Tests/examinations performed in the clinic

Prevention/guidance on:

Diet □ Smoking □ Alcohol □ Exercise □ Other □

Biomed. problems in addition to main diagnosis Number □
Psychological problems in addition to main diagnosis Number □
Social problems in addition to main diagnosis Number □

6. Referral for: Diagnostics □ Treatment □ (both boxes may be ticked)

□ Practising medical specialist, specialty:
□ Hospital outpatient clinic, specialty:
□ Admission to hospital, specialty:
□ Physiotherapy
□ External lab.

Diagnostic imaging: X-ray □ CT □ US □ Other □
□ Referral, other:

Contact to: Home care □ Health visitor □

7. Participation from practice staff:

With help □ With independent part of the consultation □ No participation □
Could the consultation have been performed by a consultation nurse/practice staff? Yes □ No □

8. Completion of consultation:

Agreed follow-up □ Wait and see (contact if needed) □ Completed without further contact □

**About the encounter**

9. To what extent do you, as a doctor, assess that the following factors played a role in the contact?

(indicate in %)

Biomedical? □□□% Psychological? □□□% Social? □□□% Don’t know□

10. In your opinion, the final diagnosis is (tick only one box):

□ a specific medical diagnosis
□ a symptom diagnosis – self-limiting
□ a symptom diagnosis – persistent (functional somatic disorder)

11. Did you, at any point, consider cancer or other serious disease (new)? Yes □ No □

Do you still have the slightest suspicion of cancer or other serious disease (new)? Yes □ No □

12. Total time spent on the contact □□ min.

Estimate the **burden** of the contact from 1 to 10 (1=easy, 10=heavy) □□
You may state the reason for the estimated burden:
