# Supplementary material for: Symptoms as the main problem: a cross- sectional study of patient experience in primary care
Source: BMC Fam Pract. 2016 Mar 10;17:29. doi: 10.1186/s12875-016-0429-8 (PMC4785648; doi:10.1186/s12875-016-0429-8)
Supplement: Additional file 2: — Patient Questionnaire. (DOCX 26 kb) [file 12875_2016_429_MOESM2_ESM.docx]

# GP Adult Consultation Questionnaire

This is a one-way translation of an original Danish survey questionnaire used for patients after their visit to a Danish general practice. The original questionnaire was designed in Teleform; the layout was different, but the items are identical. The present study builds on data from selected parts of a comprehensive questionnaire, and only items used in this study have been translated.

**The following questions concern your visit to the general practitioner on [date].**

27. Were you expectations to the visit met?

- No, not at all
- Slightly
- To some extent
- Considerably
- Completely

**Assessment of the general practitioner that you saw**

What is your assessment of the general practitioner after this visit in regard to

28. Making you feel you had time during consultations?

29. Thoroughness?

30. Physical examination of you?

31. Explaining the purpose of tests and treatments?

32. Knowing what she/he had done or told you during previous contacts?

33. Getting an appointment to suit you?

34. Getting through to the practice on the phone?

35. Waiting time in the waiting room?

36. Getting an appointment fast enough?

Response categories for each item (28-36):

- Bad
- Reasonable
- Good
- Very good
- Exceptionally good
- Don’t know/not relevant

**Your perception of your health**

37. In general, would you say your health is:

- Excellent
- Very good
- Good
- Fair
- Poor

**Activities in your daily life**

Have you because of your health been limited in the following activities? If Yes: How much?

38. MODERATE ACTIVITIES, such as moving a table, pushing a vacuum cleaner, bowling, or playing golf: or going on your bike?

- Yes, limited a lot
- Yes, limited a little
- No, not limited at all

39. Climbing SEVERAL flights of stairs:

- Yes, limited a lot
- Yes, limited a little
- No, not limited at all

During the PAST 4 weeks, have you had any of the following problems with your work or other regular activities AS A RESULT OF YOUR PHYSICAL HEALTH?

40. ACCOMPLISHED LESS than you would like:

- Yes
- No

41. I have been limited in the kind of work or other activities that I could do:

- Yes
- No

During the PAST 4 weeks, were you limited in the kind of work you do or other regular activities AS A RESULT OF ANY EMOTIONAL PROBLEMS?

42. ACCOMPLISHED LESS than you would like:

- Yes
- No

43. I have been limited in the kind of work or other activities that I could do:

- Yes
- No

44. During the PAST 4 WEEKS, how much did PAIN interfere with your normal work (including both work outside the home and housework)?

- Not at all
- A little bit
- Moderately
- Quite a bit
- Extremely

How much of the time during the PAST 4 WEEKS

45. Have you felt calm and peaceful?

- All of the time
- Most of the time
- A good bit of the time
- Some of the time
- A little of the time
- None of the time

46. Did you have a lot of energy?

- All of the time
- Most of the time
- A good bit of the time
- Some of the time
- A little of the time
- None of the time

47. Have you felt downhearted and blue?

- All of the time
- Most of the time
- A good bit of the time
- Some of the time
- A little of the time
- None of the time

48. During the PAST 4 WEEKS, how much of the time has your PHYSICAL HEALTH OR EMOTIONAL PROBLEMS interfered with your social activities (like visiting with friends, relatives, etc.)?

- All of the time
- Most of the time
- A good bit of the time
- Some of the time
- A little of the time
- None of the time

During the past 4 weeks, how much have you been bothered by:

69. Worries that there is something seriously wrong with your body?

- Not at all
- A little
- Some
- Quite a lot
- A lot
